# Neocortical grey matter distribution underlying voluntary, flexible vocalizations in chimpanzees

**DOI:** 10.1038/srep34733

**Published:** 2016-10-05

**Authors:** Serena Bianchi, Laura D. Reyes, William D. Hopkins, Jared P. Taglialatela, Chet C. Sherwood

**Affiliations:** 1 Department of Anthropology, Center for the Advanced Study of Human Paleobiology, The George Washington University, Washington, D.C., USA; 2 Department of Neurology, Icahn School of Medicine, Mount Sinai, New York, NY, USA; 3 Neuroscience Institute, Georgia State University, Atlanta, GA, USA; 4 Department of Ecology, Evolution and Organismal Biology, Kennesaw University, Kennesaw, GA, USA

## Abstract

Vocal learning is a key property of spoken language, which might also be present in nonhuman primate species, such as chimpanzees (*Pan troglodytes*), to a limited degree. While understanding the origins of vocal learning in the primate brain may help shed light on the evolution of speech and language, little is still known regarding the neurobiological correlates of vocal flexibility in nonhuman primates. The current study used voxel-based morphometry (VBM) to assess whether the cerebral cortex of captive chimpanzees that learned to voluntarily produce sounds to attract the attention of a human experimenter (attention-getting sounds) differs in grey matter distribution compared to chimpanzees that do not exhibit this behavior. It was found that chimpanzees that produce attention-getting sounds were characterized by increased grey matter in the ventrolateral prefrontal and dorsal premotor cortices. These findings suggest that the evolution of the capacity to flexibly modulate vocal output may be associated with reorganization of regions for motor control, including orofacial movements, in the primate brain.

Speech is a highly skilled behavior, which requires the synchronized movement of the respiratory, laryngeal, and orofacial muscles^1^. In contrast to most primate calls, which are thought to be innate and genetically determined^2^, speech is acquired through vocal learning and imitation of the sounds of conspecifics. In the brain, a phylogenetically older system for innate vocalizations and a more recent one for speech in humans have been described^1^,^3^. However, little is known concerning reorganization of the primate brain to accommodate the neural substrates underlying a learned, flexible vocal communication system.

Attempts to answer this question have proven challenging not only because fossil evidence relevant to understanding extinct hominin communication skills are scarce and difficult to interpret, but also because studies of nonhuman primate calls have shown only limited evidence of vocal learning^2^. As more evidence of primate communication accumulates, however, it is becoming apparent that vocal production is more flexible than it had been previously appreciated^4^.

For instance, it has been reported that, in captivity, the calls of marmosets caged together eventually developed convergence of their acoustical properties^5^. Similarly, a recent study has claimed that the acoustic structure of referential food grunts of two distinct populations of chimpanzees converged over the course of three years as a result of living together in the same zoo^6^, though some have questioned the interpretation of these findings^7^. Individual cases of great apes imitating sounds produced by a human (e.g., whistling), and controlling vocal folds movements to produce idiosyncratic communicative sounds (i.e., wookie), have also been reported^8^,^9^. Instances of vocal flexibility in nonhuman primates have also been documented in the wild. For example, distinct features in the calls of geographically contiguous populations of chimpanzees have been deemed to function as vocal “social markers” for differentiating neighboring communities^10^. There is also evidence that wild chimpanzees use alarm calls or hoots to inform ignorant conspecifics about the presence of a snake, suggesting the these sounds are produced intentionally and voluntarily^11^,^12^,^13^. Taken together, these studies suggest that some forms of learned vocal signals, possibly precursors to the extreme vocal flexibility of human speech, may be present in chimpanzees and related ape species. However, the putative neural bases of vocal, flexible communication in the nonhuman primate brain remain largely unstudied^14^,^15^.

The current study examined differences in grey matter distribution in a population of captive chimpanzees varying in their capacity to voluntary produce sounds (e.g. extended grunts, raspberries) to attract the attention of an otherwise inattentive human experimenter (attention-getting sounds — AG). Previous studies on these animals have shown that, in the presence of food, chimpanzees are more likely to produce AG sounds when a human experimenter is facing away from them compared to conditions in which the experimenter is oriented towards them^16^. Additional studies have confirmed that the likelihood of producing an AG sound when a human and food are near the focal subject is higher compared to conditions in which either only the food or human is present^17^. Thus, it appears that the production of AG sounds is under voluntary control, and is selectively used to capture the attention of an otherwise inattentive audience^18^.

Crucially, considerable individual differences in the use of AG sounds have been reported, with data showing that about 50% of the sample reliably produce these sounds while the remaining do not. While the reasons underlying these individual differences are unclear, previous research has shown that AG sounds may be socially learned from the mother, as higher rates of concordance in their production have been found between mother-offspring diads compared to chimpanzees raised in a different social group. Other factors, including age and housing conditions, do not appear to influence the production and use of AG sounds^19^,^20^.

Drawing on these intrinsic individual differences in communicative capacities, a voxel-based morphometry (VBM) analysis was conducted^21^,^22^,^23^ to assess variation in grey matter distribution in the chimpanzee brain as a function of vocal capacities. VBM is a structural neuroimaging technique that makes use of non-invasive magnetic resonance images (MRI) collected *in vivo,* and provides a whole-brain, unbiased, spatial analysis of localized differences in grey matter distribution between groups of interest. For this study we preferred a whole-brain approach to a ROI-based approach because of the limited amount of information on regions involved in the production of this atypical form of communication in chimpanzees^14^,^15^.

## Results

**Figure 1:**
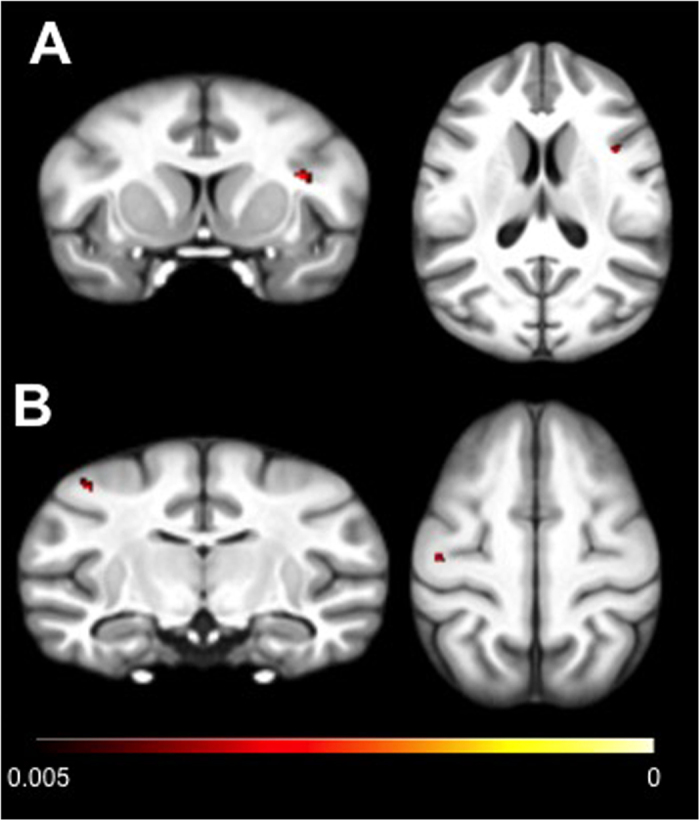
Coronal and sagittal views of the clusters showing greater grey matter density in AG+ relative to AG− chimpanzees. (**A**) ventrolateral prefrontal cortex; (**B**) dorsal premotor cortex. Clusters are superimposed onto the standard chimpanzee template.

An independent sample *t*-test assessing differences in grey matter distribution as a function of vocal phenotype in the VBM analysis revealed that chimpanzees that produce attention-getting sounds (AG+) had greater grey matter density than chimpanzees that failed to exhibit this behavior (AG−) in the right ventrolateral prefrontal cortex (VLPFC), bordering the anterior bank of the precentral gyrus, and the left dorsal premotor cortex (dPMC) (uncorrected p < 0.005) (Table 1 and Fig. 1).

The VLPFC of chimpanzees has been cytoarchitecturally mapped previously, demonstrating the localization of areas 44 and 45 on the inferior frontal gyrus^24^,^25^. The location of the ventrolateral prefrontal cluster in the current analysis was compared with that of area 44, as defined by a probabilistic map of this area in chimpanzees^24^. The cluster identified by the VBM fell within the most posterior border of area 44 mapped from a sample of 12 individuals by Schenker *et al*.^24^. Because area boundaries are known to be subject to individual variability, a more restricted map including only the portion of this region shared by at least 50% of individuals was then used. Under this more restrictive criterion, the cluster identified by the VBM fell outside the boundaries of area 44, in a location adjacent to the border with ventral premotor areas along the anterior bank of the precentral gyrus, which have been shown to elicit movements of the lips, mouth and vocal cords in chimpanzees^26^. The second cluster was located in the dorsal precentral gyrus, in the caudal part of the dorsal premotor cortex (dPMC).

## Discussion

The current study provided a whole-brain, unbiased approach to examine differences in grey matter distribution in chimpanzees that vary in their voluntarily production of attention-getting (AG) sounds in order to obtain the attention of an otherwise inattentive human experimenter. While AG sounds in this population of chimpanzees have been extensively described at the behavioral level^17^,^27^,^28^, less is known regarding their underlying neural correlates^14^,^15^,^29^. A previous PET functional neuroimaging study examining a small subset of AG+ and AG− individuals identified a number of cortical and subcortical regions that showed increased activation in association with the production of vocal and manual communicative gestures relative to a control task (i.e., grasping). These areas included the left inferior frontal gyrus, left pre- and postcentral gyrus, right parietal and cerebellar areas, and the left striatum (caudate nucleus and putamen)^15^.

A follow-up study expanding the sample size to four individuals confirmed a key role of the homologue of Broca’s area in chimpanzee communication, by showing that activation of this area was greater for the production of AG sounds and manual communicative gestures than a resting state condition in AG+ but not AG− chimpanzees^14^. At the structural level, differences in grey matter proportions have been found between chimpanzees that perform differently at a joint attention task in the anterior cingulate cortex^30^, an area that is involved in the voluntary control of vocalizations^1^,^31^, and is part of the attention system that may underlie the socio-cognitive component of these communicative gestures^32^. Consistent with previous findings, the VBM analysis performed in the current study revealed differences in grey matter distribution between AG+ and AG− in the ventral prefrontal region and premotor areas.

Given the little information currently available on the mechanisms underlying individual differences in chimpanzee vocal capacities, the possibility that motivational factors may influence the production and use of AG sounds cannot be entirely ruled out. Nonetheless, by showing structural differences, our results indicate that variation in the neural organization may indeed distinguish between AG+ and AG− chimpanzees. Moreover, as most MRI scans were collected within 5 years after initial vocal phenotype assessment, the persistence of these links between AG production and brain morphology are especially striking considering the possibility of age-related and other plastic changes in neural structure over time.

A cluster showing increased grey matter density in AG+ relative to AG− was found in the right VLPFC. Within this region, both area 44 (pars opercularis of Broca’s area) and ventral premotor cortex are key regions in the control of orofacial movements and speech processes. In humans, lesions to area 44 and the surrounding regions in the inferior frontal gyrus have been associated with forms of aphasia characterized by lack of speech fluency and agrammatism^33^,^34^,^35^. Functional neuroimaging studies have described activation of the inferior frontal gyrus in a broad variety of language tasks including word retrieval, articulation, and semantic and syntactic processes^36^,^37^,^38^.

Electrophysiological stimulations of the homologue of this area in the ventral part of the premotor cortex of macaque monkeys elicited movements of the jaw and lips, but not vocalizations^39^. Accordingly, earlier studies had ruled out a role for Broca’s area homologue in nonhuman primate vocalizations^40^. As suggested by recent studies, however, this area may be involved in the planning of voluntary, goal-directed, conditioned vocalizations in rhesus macaques^41^. In support to this view, functional studies of communicative gestures in chimpanzees indicated that the inferior frontal gyrus was associated with voluntary forms of communication, including AG sounds^14^,^15^. Functional, structural, and lesion studies have claimed that the inferior frontal gyrus, and in particular area 44 in the left hemisphere, regulates language-production functions. The cluster identified by the VBM, however, was located in the posterior part of the *right* VLPFC. In fact, although motor control of speech is typically lateralized in the left hemisphere^42^, sensorimotor control for the execution of speech and non-speech orofacial gestures has been described bilaterally^43^. Furthermore, although a linguistic role of Broca’s area has been emphasized, studies have also indicated a more general function of this area in regulating processing of hierarchical, motor sequences of behavior, regardless of their linguistic nature. Crucially, tasks involving hierarchical processing of learned motor sequences have been shown to activate both Broca’s area and its homologue in the right hemisphere^44^.

It should also be noted that in association with language functions, the right hemisphere is involved in emotional, non-propositional, pragmatic^45^, as well as prosodic (i.e., intonational) aspects of speech^46^. Since AG sounds are produced in social contexts that require some understanding of others’ intentions^17^,^30^, it is possible that a rightward increase in grey matter in AG+ relative to AG− chimpanzees reflects intrinsic differences in socio-cognitive communicative processes. Indeed, a rightward functional activation in association with the perception of conspecific calls has been described in a number of areas, including the right inferior frontal gyrus in chimpanzees^29^.

The second cluster observed in the AG+ > AG− contrast was located in the left dorsal precentral gyrus, in the caudal part of the dorsal premotor cortex (dPMC). The dPMC is extensively connected with the primary motor areas^47^,^48^, and plays a role in motor control in relation to the preparation and planning of movements in humans as well as nonhuman primates^49^,^50^,^51^. In particular, this region appears to regulate visuo-motor processes guided by arbitrary cues, a capacity that may underlie the evolution of greater flexibility over motor control by “permitting the choice of an action based on the prevailing context” rather than on spatial constraints^52^. Because arbitrary mapping is a key property of language (e.g., sound-meaning), it has been suggested that control over learned guided actions may have provided one motor preadaptation for language^52^.

Accordingly, a recently proposed model of language processing posits that dorsal premotor areas are part of a dorsal system of language production that provides a phonological-articulatory interface^53^, and potentially, an integration area for vocal learning. Interestingly, a recent study measuring activity-induced cFos expression in marmosets found that stronger labeling of the dorsal prefrontal cortex was associated with call production, while expression in the ventral premotor areas was associated with auditory perception of conspecifics calls^54^.

While a role for dPMC in the evolution of motor control for language is plausible, reasons underlying purported differences in grey matter distribution of this area between AG+ and AG− chimpanzees should be further assessed. Indeed, the cluster identified by the VBM was located slightly dorsal to the “knob”, or hand region of the precentral gyrus^55^. As such, this location is compatible with an area adjacent to the motor somatotopic representation of the upper limbs^26^,^56^. Numerous lesion, electrophysiological, and functional neuroimaging studies in macaque monkeys as well as humans have shown that the dorsal premotor cortex is a key region underlying motor control of the arm, including movements directed towards a target, such as grasping and reaching^51^,^57^. This may be relevant because in combination with vocal signals like AG sounds, chimpanzees have been shown to use a number of communicative manual gestures, such as extending their arm in a food begging gesture, and pointing with either the open hand or the finger to attract the attention of the experimenter^19^,^58^.

Furthermore, although most chimpanzees engage in manual gestures to some extent, those producing AG sounds appear to more often throw objects to attract the attention of the experimenter^59^. Accordingly, a study comparing the neuroanatomical and cognitive correlates of throwing capacity in chimpanzees indicated that individuals that throw objects are characterized by greater white matter to grey matter volume in the hand ‘knob’ region compared to those that engage less in this behavior^59^. Crucially, “throwers” also showed increased socio-communicative skills as compared to “non-throwers”, while no differences in measures of physical cognition distinguished the two groups^59^.

Thus, it is possible that a trend towards grey matter differences in the dorsal premotor cortex of AG+ and AG− chimpanzees reflects intrinsic motor capacities for gestural movements of the arm and hand that are co-produced with AG sounds, such as manual gestures, throwing, clapping, and using objects (e.g., making noises) in order to attract the attention of the experimenter. A left lateralization of the results is also consistent with previous reports of population level right-handedness of chimpanzees for gestural communication and throwing^59^,^60^. Whether and how vocal and gestural capacities interact to produce greater flexibility for communication in chimpanzees, however, remains to be ascertained.

Our findings revealed localized increases in grey matter in prefrontal and premotor areas, thus suggesting that intrinsic differences in the capacity to communicate flexibly may be associated with variation in the plasticity of areas of the cerebral cortex supporting motor control of orofacial and gestural movements.

Comparative studies have shown that relative to Old World monkeys, the great apes have increased neuropil space for neuronal integration in the orofacial representation of the primary motor cortex, possibly as a result of selection for increased control of the orofacial communicative gestures^61^. Indeed, the great apes produce many facial expressions in communicative contexts, which may be associated with vocalizations, but also often lack in vocal content (e.g., pouting)^62^.

If and to what extent human language capacities started in a vocal or gestural modality is a long-standing, and hotly debated question^63^,^64^. By showing that flexible communication in chimpanzees involves morphological differences in cortical regions important for both vocal/orofacial movements as well as manual gestures, our results encourage further study of the co-evolution of multi-modal communication and the brain. How brain reorganization in human evolution led to the acoustic-vocal modality of speech as the preferred, while not only, form of communication of our species, remains to be fully understood.

## Methods

### Sample

All the chimpanzees examined in the current study were housed at the Yerkes National Primate Research Center (YNRC) in Atlanta, GA, USA, in accordance with institutional guidelines. Most of these individuals had taken part in previous behavioral studies on tool-use, handedness, and communication (e.g. refs 14,20,28,65 and 66).

The current study included sixty-nine chimpanzees, of which 34 were classified as individuals that did not produce attention getting sounds (AG−) and 35 as individuals that produced attention-getting sounds (AG+) (see below for a description of their vocal assessment). The AG− group was comprised of 7 males, and 27 females, while the AG+ group consisted of 15 males, and 20 females. All individuals were adults, and no significant differences were found between AG groups as function of age (t_1,67_ = 1.31, *p* = 0.19).

### Behavioral assessment of vocal phenotype

Chimpanzees were grouped according to their vocal phenotype, where AG+ indicated individuals that produce attention-getting sounds, and AG− denoted individuals who did NOT reliably show this behavior. The procedure for vocal assessment has been described elsewhere^17^,^20^,^28^. Briefly, each chimpanzee received 6, 30-sec test trials. During each test trial, food was placed outside of the subjects’ home enclosure and a human experimenter would approach the subject. On three trials, the experimenter was oriented away from the chimpanzees while on the remaining 3 trials the human was oriented toward the subject. All the communicative behaviors directed toward the experimenter in each condition were recorded. AG sounds were defined as audible sounds that are distinct from the naturally occurring chimpanzee calls (e.g., food calls, alarms^28^), and included “raspberries”, extended grunts and other idiosyncratic sounds. If the chimpanzees produced at least one AG sound during any of the 6 trials, they were classified as AG+ while all others were classified as AG−.

### MRI scanning procedure and preprocessing

Magnetic Resonance Image (MRI) data acquisition was performed at the imaging facilities of the YNPRC following institutional guidelines. The procedure has been described in detail elsewhere (e.g. ref. 67). Briefly, each individual was first immobilized with a telazol injection (2–6 mg/kg), and then anesthetized with propofol (10 mg/kg/h). The total time, including scan acquisition (40–50 mins) and the transport to and from the MRI facility, was about 2 hours. A 3.0 Tesla scanner (Siemens Trio; Siemens Medical Solutions Inc., Malvern, PA, USA) was used. After the scanning was concluded, the chimpanzees were housed in a separate cage for 6–12 hours until while they recovered from the anesthesia.

MRI scans were preprocessed using FSL software and included skull stripping and image normalization using the BET function (http://fsl.fmrib.ox.ac.uk/fsl/fslwiki/BET). MRIs of the individuals examined in the current study have been previously employed in studies investigating structural features of the chimpanzee brain (e.g. ref. 67), and their correlations with known behavioral traits such as handedness (e.g. refs 30 and 59).

### VBM analysis

Whole-brain comparisons between AG− and AG+ chimpanzees were performed on MRI structural data with the VBM tool available in FSL software (http://fsl.fmrib.ox.ac.uk/fsl/fslwiki/FSLVBM). An optimized VBM protocol^22^ adapted to the chimpanzee brain was used. Structural images were first brain-extracted, and grey matter-segmented. All native grey matter images were then non-linearly registered to a chimpanzee standard template^68^, and modulated to correct for local expansion (or contraction) due to the non-linear component of the spatial transformation (http://fsl.fmrib.ox.ac.uk/fsl/fslwiki/FSLVBM). The modulated grey matter images were then smoothed with an isotropic Gaussian kernel with a sigma of 2 mm, FHWM = ~4.7 mm. To assess group-level differences between AG+ and AG− chimpanzees, voxel-wise GLM was applied using permutation-based non-parametric testing, with a TFCE cluster-based method (fsl.fmrib.ox.ac.uk/fsl/fslwiki/FSLVBM). Results from the comparisons between AG+ and AG− chimpanzees were thresholded at p = 0.005 (uncorrected), and only clusters with *t* > 2.00 and a size >20 voxels were reported. In the analyses, sex, age and overall brain volume were included as covariates, to rule out effects of inter-individual differences due to potentially confounding variables. The data of this study will be made available at chimpanzeebrain.org.

## Additional Information

**How to cite this article**: Bianchi, S. *et al*. Neocortical grey matter distribution underlying voluntary, flexible vocalizations in chimpanzees. *Sci. Rep.* **6**, 34733; doi: 10.1038/srep34733 (2016).

## Figures and Tables

**Table 1:** VBM results AG+ > AG− including the coordinates of reported clusters and t statistics at p-value = 0.005 (uncorrected).

Region	Coordinates (X, Y, Z)	Cluster size (voxels #)	t
Right VLPFC	126,123, 61	40	3.40
Left dPMC	55, 100,78	28	2.72

## References

[b1] Simonyan K. & Horwitz B. Laryngeal motor cortex and control of speech in humans. Neuroscientist 17, 197–208 (2011). 21362688 10.1177/1073858410386727 PMC3077440

[b2] Egnor S. E. R. & Hauser M. D. A paradox in the evolution of primate vocal learning. Trends Neurosci. 27, 649–654 (2004). 15474164 10.1016/j.tins.2004.08.009

[b3] Jurgens U. The neural control of vocalization in mammals: a review. J. Voice 23, 1–10 (2009). 18207362 10.1016/j.jvoice.2007.07.005

[b4] Petkov C. I. & Jarvis E. D. Birds, primates, and spoken language origins: behavioral phenotypes and neurobiological substrates. Front. Evol. Neurosci. 4, 12 (2012). 22912615 10.3389/fnevo.2012.00012 PMC3419981

[b5] Snowdon C. T. Plasticity of Communication in Nonhuman Primates. Adv. Stud. Behav. 40, 239–276 (Elsevier Inc., 2009).

[b6] Watson S. K. . Vocal learning in the functionally referential food grunts of chimpanzees. Curr. Biol. 25, 495–499 (2015). 25660548 10.1016/j.cub.2014.12.032

[b7] Fischer J., Wheeler B. C. & Higham J. P. Is there any evidence for vocal learning in chimpanzee food calls? Curr. Biol. 25, R1028–R1029 (2015). 26528740 10.1016/j.cub.2015.09.010

[b8] Wich S. A. . A case of spontaneous acquisition of a human sound by an orangutan. Primates 50, 56–64 (2009). 19052691 10.1007/s10329-008-0117-y

[b9] Lameira A. R., Hardus M. E., Mielke A., Wich S. A. & Shumaker R. W. Vocal fold control beyond thespecies-speci c repertoire in anorang-utan. Sci. Rep. 1–10, doi: 10.1038/srep30315 (2016). PMC4962094 27461756

[b10] Crockford C., Herbinger I., Vigilant L. & Boesch C. Wild chimpanzees produce group‐specific calls: a case for vocal learning? Ethology 110, 221–243 (2004).

[b11] Crockford C., Wittig R. M. & Zuberbühler K. An intentional vocalization draws others’ attention: a playback experiment with wild chimpanzees. Anim. Cogn. 18, 581–591 (2015). 25537683 10.1007/s10071-014-0827-z

[b12] Crockford C., Wittig R. M., Mundry R. & Zuberbühler K. Wild chimpanzees inform ignorant group members of danger. Curr. Biol. 22, 142–146 (2012). 22209531 10.1016/j.cub.2011.11.053

[b13] Schel A. M., Townsend S. W., Machanda Z., Zuberbühler K. & Slocombe K. E. Chimpanzee alarm call production meets key criteria for intentionality. PLoS ONE 8, e76674 (2013). 24146908 10.1371/journal.pone.0076674 PMC3797826

[b14] Taglialatela J. P., Russell J. L., Schaeffer J. A. & Hopkins W. D. Chimpanzee vocal signaling points to a multimodal origin of human language. PLoS ONE 6, e18852 (2011). 21533079 10.1371/journal.pone.0018852 PMC3080370

[b15] Taglialatela J. P., Russell J. L., Schaeffer J. A. & Hopkins W. D. Communicative signaling activates ‘Broca’s’ homolog in chimpanzees. Curr. Biol. 18, 343–348 (2008). 18308569 10.1016/j.cub.2008.01.049 PMC2665181

[b16] Leavens D. A., Hostetter A. B., Wesley M. J. & Hopkins W. D. Tactical use of unimodal and bimodal communication by chimpanzees, Pan troglodytes. Anim. Behav. 67, 467–476 (2004).

[b17] Hopkins W. D., Taglialatela J. & Leavens D. A. Chimpanzees differentially produce novel vocalizations to capture the attention of a human. Anim. Behav. 73, 281–286 (2007). 17389908 10.1016/j.anbehav.2006.08.004 PMC1832264

[b18] Hopkins W. D., Taglialatela J. P., Leavens D. A. & Vauclair J. In Primate communication and human language (eds Vilain A., Schwartz J.-L., Abry C. & Vauclair J.) 206–226 (John Benjamins Publishing) (2011).

[b19] Leavens D. A., Hopkins W. D. & Thomas R. K. Referential communication by chimpanzees (*Pan troglodytes*). J. Comp. Psychol. 118, 48–57 (2004). 15008672 10.1037/0735-7036.118.1.48

[b20] Taglialatela J. P., Reamer L., Schapiro S. J. & Hopkins W. D. Social learning of a communicative signal in captive chimpanzees. Biol. Lett. 8, 498–501 (2012). 22438489 10.1098/rsbl.2012.0113 PMC3391466

[b21] Ashburner J. & Friston K. J. Why voxel-based morphometry should be used. NeuroImage 14, 1238–1243 (2001). 11707080 10.1006/nimg.2001.0961

[b22] Good C. D. . A voxel-based morphometric study of ageing in 465 normal adult human brains. NeuroImage 14, 21–36 (2001). 11525331 10.1006/nimg.2001.0786

[b23] Smith S. M. . Advances in functional and structural MR image analysis and implementation as FSL. NeuroImage 23 Suppl 1, S208–S219 (2004). 15501092 10.1016/j.neuroimage.2004.07.051

[b24] Schenker N. M. . Broca’s area homologue in chimpanzees (*Pan troglodytes*): probabilistic mapping, asymmetry, and comparison to humans. Cereb. Cortex 20, 730–742 (2010). 19620620 10.1093/cercor/bhp138 PMC2820707

[b25] Sherwood C. C., Broadfield D. C., Holloway R. L., Gannon P. J. & Hof P. R. Variability of Broca’s area homologue in African great apes: Implications for language evolution. Anat. Rec. 271A, 276–285 (2003). 10.1002/ar.a.10046 12629670

[b26] Leyton A. S. F. & Sherrington C. S. Observations on the excitable cortex of the chimpanzee, orangutan, and gorilla. Exp. Physiol. 11, 135–222 (1917).

[b27] Reynolds Losin E. A., Russell J. L., Freeman H., Meguerditchian A. & Hopkins W. D. Left hemisphere specialization for oro-facial movements of learned vocal signals by captive chimpanzees. PLoS ONE 3, e2529 (2008). 18575610 10.1371/journal.pone.0002529 PMC2429967

[b28] Russell J. L., McIntyre J. M., Hopkins W. D. & Taglialatela J. P. Vocal learning of a communicative signal in captive chimpanzees, *Pan troglodytes*. Brain Lang. 127, 520–525 (2013). 24144730 10.1016/j.bandl.2013.09.009 PMC3982915

[b29] Taglialatela J. P., Russell J. L., Schaeffer J. A. & Hopkins W. D. Visualizing vocal perception in the chimpanzee brain. Cereb. Cortex 19, 1151–1157 (2008). 18787228 10.1093/cercor/bhn157 PMC2665158

[b30] Hopkins W. D. & Taglialatela J. P. Initiation of joint attention is associated with morphometric variation in the anterior cingulate cortex of chimpanzees (*Pan troglodytes*). Am. J. Primatol. 75, 441–449 (2013). 23300067 10.1002/ajp.22120 PMC3609881

[b31] Jurgens U. Neural pathways underlying vocal control. Neurosci. Biobehav. Rev. 26, 235–258 (2002). 11856561 10.1016/s0149-7634(01)00068-9

[b32] Paus T. Primate anterior cingulate cortex: where motor control, drive and cognition interface. Nat. Rev. Neurosci. 2, 417–424 (2001). 11389475 10.1038/35077500

[b33] Kent R. D. Research on speech motor control and its disorders: a review and prospective. J. Commun. Disord. 33, 391–427 (2000). 11081787 10.1016/s0021-9924(00)00023-x

[b34] Dronkers N. F., Plaisant O., Iba-Zizen M. T. & Cabanis E. A. Paul Broca’s historic cases: high resolution MR imaging of the brains of Leborgne and Lelong. Brain 130, 1432–1441 (2007). 17405763 10.1093/brain/awm042

[b35] Damasio A. R. Aphasia. N Engl J Med 326, 531–539 (1992). 1732792 10.1056/NEJM199202203260806

[b36] Price C. J. The anatomy of language: contributions from functional neuroimaging. J. Anat. 197 Pt 3, 335–359 (2000). 11117622 10.1046/j.1469-7580.2000.19730335.x PMC1468137

[b37] Price C. J. The anatomy of language: a review of 100 fMRI studies published in 2009. Ann. N. Y. Acad. Sci. 1191, 62–88 (2010). 20392276 10.1111/j.1749-6632.2010.05444.x

[b38] Friederici A. D., Kotz S. A., Werheid K., Hein G. & Cramon, von D. Y. Syntactic comprehension in Parkinson’s disease: Investigating early automatic and late integrational processes using event-related brain potentials. Neuropsychology 17, 133–142 (2003). 12597082

[b39] Petrides M., Cadoret G. & Mackey S. Orofacial somatomotor responses in the macaque monkey homologue of Broca’s area. Nature 435, 1235–1238 (2005). 15988526 10.1038/nature03628

[b40] Jurgens U., Lu C. L. & Quondamatteo F. C-fos expression during vocal mobbing in the new world monkey Saguinus fuscicollis. Eur. J. Neurosci. 8, 2–10 (1996). 8713445 10.1111/j.1460-9568.1996.tb01162.x

[b41] Hage S. R. & Nieder A. Single neurons in monkey prefrontal cortex encode volitional initiation of vocalizations. Nat. Commun. 4, 2409 (2013). 24008252 10.1038/ncomms3409

[b42] Simonyan K., Herscovitch P. & Horwitz B. Speech-induced striatal dopamine release is left lateralized and coupled to functional striatal circuits in healthy humans: A combined PET, fMRI and DTI study. NeuroImage 70, 21–32 (2013). 23277111 10.1016/j.neuroimage.2012.12.042 PMC3580021

[b43] Grabski K. . Functional MRI assessment of orofacial articulators: neural correlates of lip, jaw, larynx, and tongue movements. Hum. Brain Mapp. 33, 2306–2321 (2012). 21826760 10.1002/hbm.21363 PMC6870116

[b44] Koechlin E. & Jubault T. Broca’s area and the hierarchical organization of human behavior. Neuron 50, 963–974 (2006). 16772176 10.1016/j.neuron.2006.05.017

[b45] Lindell A. K. In your right mind: right hemisphere contributions to language processing and production. Neuropsychol. Rev. 16, 131–148 (2006). 17109238 10.1007/s11065-006-9011-9

[b46] Wildgruber D. . Identification of emotional intonation evaluated by fMRI. NeuroImage 24, 1233–1241 (2005). 15670701 10.1016/j.neuroimage.2004.10.034

[b47] Barbas H. & Pandya D. N. Architecture and frontal cortical connections of the premotor cortex (area 6) in the rhesus monkey. J. Comp. Neurol. 256, 211–228 (1987). 3558879 10.1002/cne.902560203

[b48] Dum R. P. & Strick P. L. Motor areas in the frontal lobe of the primate. Physiol. Behav. 77, 677–682 (2002). 12527018 10.1016/s0031-9384(02)00929-0

[b49] Wise S. P., Boussaoud D., Johnson P. B. & Caminiti R. Premotor and parietal cortex: corticocortical connectivity and combinatorial computations. Annu. Rev. Neurosci. 20, 25–42 (1997). 9056706 10.1146/annurev.neuro.20.1.25

[b50] Chouinard P. A. & Paus T. The primary motor and premotor areas of the human cerebral cortex. Neuroscientist 12, 143–152 (2006). 16514011 10.1177/1073858405284255

[b51] Davare M., Andres M., Cosnard G., Thonnard J.-L. & Olivier E. Dissociating the role of ventral and dorsal premotor cortex in precision grasping. J. Neurosci. 26, 2260–2268 (2006). 16495453 10.1523/JNEUROSCI.3386-05.2006 PMC6674806

[b52] Wise S. P. & Murray E. A. Arbitrary associations between antecedents and actions. Trends Neurosci. 23, 271–276 (2000). 10838597 10.1016/s0166-2236(00)01570-8

[b53] Hickok G. & Poeppel D. The cortical organization of speech processing. Nat. Rev. Neurosci. 8, 393–402 (2007). 17431404 10.1038/nrn2113

[b54] Miller C. T., Dimauro A., Pistorio A., Hendry S. & Wang X. Vocalization induced CFos expression in marmoset cortex. Front. Integr. Neurosci. 4, 128 (2010). 21179582 10.3389/fnint.2010.00128 PMC3004388

[b55] Hopkins W. D., Taglialatela J. P., Russell J. L., Nir T. M. & Schaeffer J. Cortical representation of lateralized grasping in chimpanzees (*Pan troglodytes*): a combined MRI and PET study. PLoS ONE 5, e13383 (2010). 20967216 10.1371/journal.pone.0013383 PMC2954174

[b56] Meier J. D., Aflalo T. N., Kastner S. & Graziano M. S. A. Complex organization of human primary motor cortex: a high-resolution fMRI study. J. Neurophysiol. 100, 1800–1812 (2008). 18684903 10.1152/jn.90531.2008 PMC2576195

[b57] Turella L. & Lingnau A. Neural correlates of grasping. Front. Hum. Neurosci. 8, 686 (2014). 25249960 10.3389/fnhum.2014.00686 PMC4158794

[b58] Hostetter A. B., Cantero M. & Hopkins W. D. Differential use of vocal and gestural communication by chimpanzees (*Pan troglodytes*) in response to the attentional status of a human (*Homo sapiens*). J. Comp. Psychol. 115, 337–343 (2001). 11824896 10.1037//0735-7036.115.4.337 PMC2080764

[b59] Hopkins W. D., Russell J. L. & Schaeffer J. A. The neural and cognitive correlates of aimed throwing in chimpanzees: a magnetic resonance image and behavioural study on a unique form of social tool use. Philos. Trans. R. Soc. Lond., B, Biol. Sci. 367, 37–47 (2012). 22106425 10.1098/rstb.2011.0195 PMC3223792

[b60] Hopkins W. D., Russell J. L., Cantalupo C., Freeman H. & Schapiro S. J. Factors influencing the prevalence and handedness for throwing in captive chimpanzees (*Pan troglodytes*). J. Comp. Psychol. 119, 363–370 (2005). 16366769 10.1037/0735-7036.119.4.363 PMC2680150

[b61] Sherwood C. C., Holloway R. L., Erwin J. M. & Hof P. R. Cortical orofacial motor representation in Old World Monkeys, great apes, and humans. Brain Behav. Evol. 63, 82–106 (2004). 14685003 10.1159/000075673

[b62] Pollick A. S. & de Waal F. B. M. Ape gestures and language evolution. Proc. Natl. Acad. Sci. USA 104, 8184–8189 (2007). 17470779 10.1073/pnas.0702624104 PMC1876592

[b63] MacNeilage P. F. The frame/content theory of evolution of speech production. Behav. Brain. Sci. 21, 499–511 (1998). 10097020 10.1017/s0140525x98001265

[b64] Corballis M. C. From mouth to hand: gesture, speech, and the evolution of right-handedness. Behav. Brain Sci. 26, 199–208 (2003). 14621511 10.1017/s0140525x03000062

[b65] Hopkins W. D., Russell J. L., Schaeffer J. A., Gardner M. & Schapiro S. J. Handedness for tool use in captive chimpanzees (*Pan troglodytes*): Sex differences, performance, heritability and comparison to the wild. Behaviour 146, 1463–1483 (2009). 20221316 10.1163/156853909X441005 PMC2835370

[b66] Hopkins W. D., Reamer L., Mareno M. C. & Schapiro S. J. Genetic basis in motor skill and hand preference for tool use in chimpanzees (*Pan troglodytes*). Proc. Biol. Sci. 282, 20141223 (2015). 25520351 10.1098/rspb.2014.1223 PMC4298198

[b67] Hopkins W. D. . Gray matter asymmetries in chimpanzees as revealed by voxel-based morphometry. NeuroImage 42, 491–497 (2008). 18586523 10.1016/j.neuroimage.2008.05.014 PMC2569890

[b68] Hopkins W. D. & Avants B. B. Regional and hemispheric variation in cortical thickness in chimpanzees (*Pan troglodytes*). J. Neurosci. 33, 5241–5248 (2013). 23516289 10.1523/JNEUROSCI.2996-12.2013 PMC3643894

